# Common Pathogenetic Mechanisms Underlying Aging and Tumor and Means of Interventions

**DOI:** 10.14336/AD.2021.1208

**Published:** 2022-07-11

**Authors:** Weiyi Shen, Jiamin He, Tongyao Hou, Jianmin Si, Shujie Chen

**Affiliations:** ^1^Department of Gastroenterology, Sir Run Run Shaw Hospital, Zhejiang University School of Medicine, Hangzhou 310016, Zhejiang, China.; ^2^Institute of Gastroenterology, Zhejiang University, Hangzhou 310016, Zhejiang, China.; ^3^Prevention and Treatment Research Center for Senescent Disease, Zhejiang University School of Medicine, Zhejiang, China

**Keywords:** aging, cancer, similarities, pathogenetic mechanisms, interventions

## Abstract

Recently, there has been an increase in the incidence of malignant tumors among the older population. Moreover, there is an association between aging and cancer. During the process of senescence, the human body suffers from a series of imbalances, which have been shown to further accelerate aging, trigger tumorigenesis, and facilitate cancer progression. Therefore, exploring the junctions of aging and cancer and searching for novel methods to restore the junctions is of great importance to intervene against aging-related cancers. In this review, we have identified the underlying pathogenetic mechanisms of aging-related cancers by comparing alterations in the human body caused by aging and the factors that trigger cancers. We found that the common mechanisms of aging and cancer include cellular senescence, alterations in proteostasis, microbiota disorders (decreased probiotics and increased pernicious bacteria), persistent chronic inflammation, extensive immunosenescence, inordinate energy metabolism, altered material metabolism, endocrine disorders, altered genetic expression, and epigenetic modification. Furthermore, we have proposed that aging and cancer have common means of intervention, including novel uses of common medicine (metformin, resveratrol, and rapamycin), dietary restriction, and artificial microbiota intervention or selectively replenishing scarce metabolites. In addition, we have summarized the research progress of each intervention and revealed their bidirectional effects on cancer progression to compare their reliability and feasibility. Therefore, the study findings provide vital information for advanced research studies on age-related cancers. However, there is a need for further optimization of the described methods and more suitable methods for complicated clinical practices. In conclusion, targeting aging may have potential therapeutic effects on aging-related cancers.

Aging of the human population is gradually becoming an important global issue. According to the United Nations, the global human population aged >60 years will increase by two-fold by 2050 compared to that in 2000 [1]. Aging of the human population is gradually becoming an important global issue. According to the United Nations, the global human population aged >60 years will increase by two-fold by 2050 compared to that in 2000 [2]. Therefore, aging causes severe stress on the human health system. Therefore, there is an urgent need to improve the health of this population. To explore the mechanism of aging, several studies have found several common hallmarks of aging in different individuals, such as genomic alteration, epigenetic changes, mitochondrial damage, and cellular senescence. The findings of previous studies have provided clues to further explore the mechanisms of anti-aging or alleviating aging-related damage and cancer [3].

Recently, the high rates of morbidity and mortality caused by various cancers have made it a much-researched topic. According to statistics, the incidence of cancer among humans is 11-fold higher in individuals aged >65 years than in individuals aged < 65 years. Furthermore, most cancers are diagnosed in people aged >55 years [4]. Currently, the gap in the incidence of cancer between young and older individuals has increased compared to that described earlier. In some cancers, aging is a factor that leads to malignant tumors with a poorer prognosis; hence, various cancers seem to have a close relationship with aging [5,6].

A recent study performed by Gomes at al. revealed that aging-mediated metabolic reconstitution, especially accumulation of methylmalonic acid, can form an internal environment that facilitates cancer cell growth and progression [7]. These findings further confirmed the existence of a mysterious intersection between aging and cancer. Therefore, exploring the pathogenesis of aging-induced cancer is of great importance. In this study, the junctions of aging and cancer were addressed by comparing various research findings. The study results indicate that delaying aging may be a promising intervention against aging-related cancers.

## Aging and cancer have common pathogenetic mechanisms

**Figure 1.**
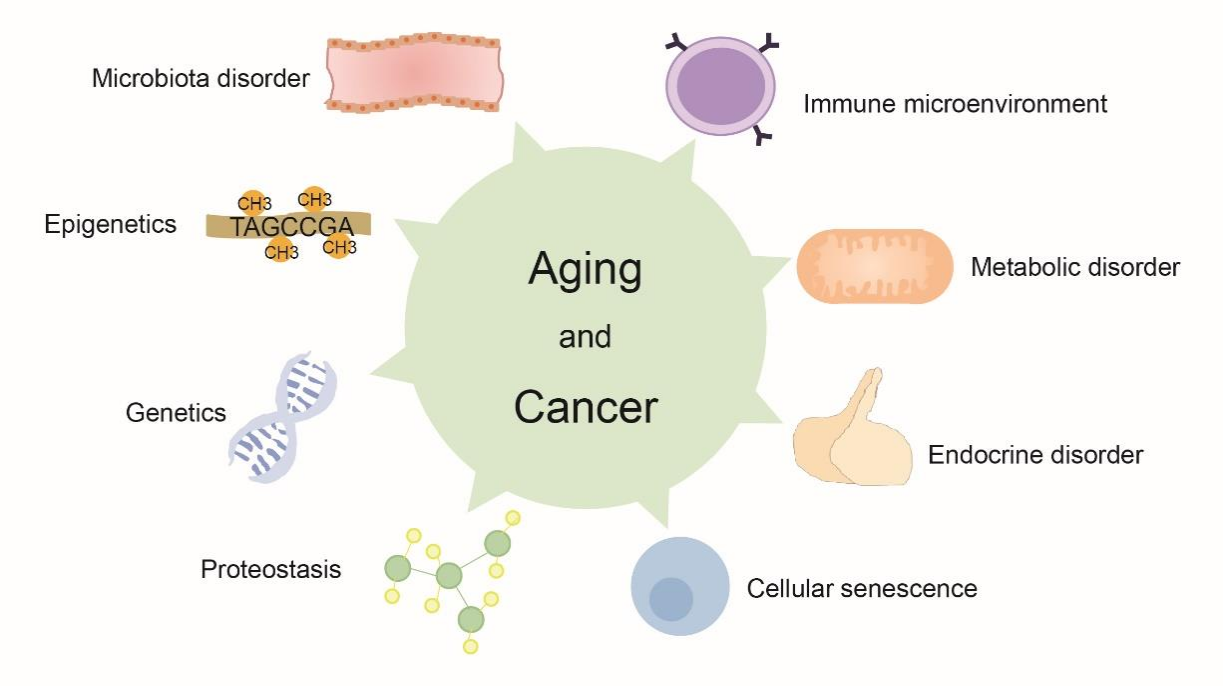
Common pathogenetic mechanisms between aging and cancer.

The mechanisms of the occurrence and development of aging and cancer are being researched since the past few decades, and several results have been achieved. These studies have revealed several mechanisms involved in the occurrence and/or development of both aging and cancer. For instance, studies have reported that cellular senescence significantly diminishes in malignant tumors, enhanced ubiquitin-proteasome and lysosome-autophagy systems prolong lifespan but promote cancer progression, and alterations in the systemic immune microenvironment mediated by aging push further demic recession and cancer appearance. In addition, intestinal flora, local or systemic metabolism, and hormones change with age, which in turn affects the longevity of human, drives tumorigenesis, and causes cancer deterioration in health simultaneously. Moreover, aging and cancer are regulated by the expression of similar genes. Therefore, we aimed to precisely describe the common mechanisms involved in the occurrence and development of both aging and cancer (Fig. 1,Table 1, and Table 2).

## Cellular senescence: What happens in cancer?

Cellular senescence refers to the permanent proliferative arrest of cells subjected to stressors. It can be induced by various physiological and pathological factors, such as telomere erosion, oxidative stress, and activation of oncogenes [8].

In cancer cells, powerful telomerase activity leads to attenuated telomere erosion and delayed cellular senescence. Therefore, antitelomerase therapy can be an important therapeutic intervention for malignant growth and can induce cell death. Notably, cancers that retain wild-type *p53*, a well-known anti-oncogene, can falsify the response to antitelomerase therapies, which only trigger proliferative arrest rather than inducing cell death. Furthermore, this change results in prolonged treatment and increased drug resistance [9].

**Table 1:** Common mechanism between aging and cancer.

Mechanism	Alteration during aging	Effect on aging	Effect on cancer
**Proteostasis**			
**Chaperones**	Decrease	Delay	Promote
**Ubiquitin-proteasome**	Decrease	Delay	Promote
**Lysosome-autophagy**	Decrease	Delay	Promote
**Microbiota disorder**			
**Akkermansia**	Decrease	Delay	Inhibit
**Bifidobacterium**	Decrease	Delay	Inhibit
**Escherichia coli**	Increase	/	Promote
**Bacteroides fragilis**	Increase	/	Promote
**Immune microenvironment**			
**Inflammation**	Increase	Accelerate	Promote
**Immunosenescence**	Increase	/	Promote
**Metabolic disorder**			
**Energy metabolism**			
**NAD+**	Decrease	Delay	Promote
**Mitochondrial damage**	Increase	Accelerate	Promote
**Substance metabolism**			
**Methylmalonic acid**	Increase	/	Promote
**Quinolinate**	Increase	/	Promote
**Phosphoenolpyruvate**	Increase	/	Inhibit
**α-ketoglutarate**	Decrease	Delay	Bidirectional
**Endocrine disorder**			
**TSH**	Decrease	Delay	Promote
**GH/IGF-1**	Decrease	Accelerate	Promote
**FSH/LH**	Decrease	Delay	Promote
**Genetics**			
**Klotho**	Low expression	Delay	Inhibit
**AUF1**	/	Delay	Bidirectional
**SIRT1**	Low expression	Delay	Bidirectional
**P16**	High expression	Accelerate	Inhibit

NAD+, nicotinamide adenine dinucleotide; TSH, thyroid stimulating hormone; GH, growth hormone; IGF-1, insulin-like growth factor I; FSH, follicle-stimulating hormone; LH, luteinizing hormone.

The life and death of cells is largely determined by their redox status. Oxidative stress is a cause of cellular senescence in normal cells. In malignant cells, although excessive proliferation results in higher generation of reactive oxygen species (ROS), the cells can realign their redox status and enhance their antioxidant ability, thereby avoiding reaching the ROS thresholds that trigger cellular senescence. Moreover, the cells can also optimize ROS-mediated DNA damage and subsequent malignant proliferation [10].

**Table 2:** Common phenomena between aging and cancer.

Phenomenon	Alteration during aging	Alteration in cancer
**Cellular senescence**	Increase	Increase (premalignant tumor) Decrease (malignant tumors)
**Epigenetics**		
**DNA Methylation age**	Accelerate	Accelerate
**Genomic imprinting**	Increase/Decrease	Increase/Decrease
**Gene silencing**	Loss	Loss

**Figure 2.**
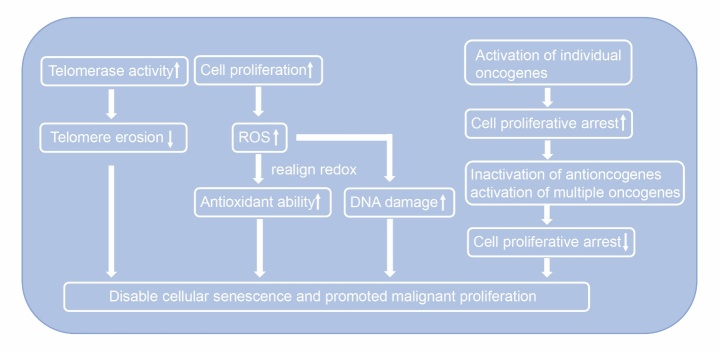
**Disabling cellular senescence in cancer**. 1. Powerful telomerase activity attenuates telomere erosion; 2. Realigning redox improves antioxidant capacity; 3. Although activation of individual oncogenes triggers cell proliferative arrest in the early stage, subsequent inactivation of anti-oncogenes and activation of multiple oncogenes restores cell proliferation.

In premalignant tumors, activation of oncogenes, such as *HRASG12V* and *EGFR*, leads to permanent cell proliferative arrest and inhibits cancer progression [11,12]. In other words, cellular senescence serves as a protective mechanism and extensively exists in the precancerous stage. However, during the stage of tumor progression and transformation to malignancy, inactivation of anti-oncogenes and common overexpression of other oncogenes disables the process of cellular senescence [13] (Fig. 2).

## Alterations in proteostasis links aging with cancer

Chaperones, ubiquitin-proteasome, and lysosome-autophagy systems are important pillars of proteostasis. ATP-dependent chaperones are significantly inhibited during the aging process because of damage to energy metabolism. A research study on the nervous system revealed that 32% ATP-dependent chaperones were downregulated in the aged brain [14]. In addition, there was also a sharp decrease in the chaperones that play a role in target recognition [15]. In older individuals, a marked decline in autophagic function has also been shown in various species [16,17], which may be due to a decline in the expression of *Atg* and *Sirtuin 1* or hyperactivation of TORC1 [18]. Moreover, repression of proteasome activity has been observed in the early stages of various organic aging processes, such as aging of the brain and skin [19,20].

In contrast, alterations in proteostasis also influence the process of aging. In yeast, increasing proteasome capacity by upregulating the transcription factor Rpn4 can prolong their replicative lifespan [21]. In Drosophila melanogaster, upregulation of Rpn11 and DmPI31, gamma irradiation, and other processes have been shown to extend the lifespan by activating the proteasome [22-24]. In mice, the phenomenon of delaying aging by upregulation of IGF-1 or dietary intervention has been shown to be closely related to proteasome activation [25,26]. In humans, powerful proteasome activity has also been observed in centenarians [27]. Similarly, most pro-longevity methods have been shown to enhance autophagic function [28]. According to Krøll et al., RNA chaperones are involved in cell immortalization [29]. Furthermore, Ito et al. reported that improving mitochondrial chaperones could lead to longevity in elegans [30].

However, although enhanced proteasome capacity and autophagic function can extend the lifespan, it seems to promote cancer progression. For instance, the proteasome activator REGγ can enhance the transforming growth factor (TGF)-β pathway and result in lung cancer metastasis [31]. Tribble homolog 2 promotes ubiquitin degradation and weakens oxidative damage by upregulating proteasome activity, which promotes the progression of liver cancer [32]. Furthermore, *Fusobacterium nucleatum* has been shown to induce chemoresistance by activating autophagy in colorectal cancer (CRC) [33]. Autophagy also triggers immune evasion by improving MHC-I degradation in pancreatic malignant tumors [34]. In addition, cachexia, ubiquitin-proteasome, and lysosome-autophagy systems are significantly activated in gastric cancer [35]. Moreover, heat shock proteins (HSPs), especially HSP90 and HSP70, play an important role in folding and transporting pivotal cancer proteins and are indispensable factors in cancer aggressiveness [36].

In conclusion, it was evident that powerful ubiquitin-proteasome and lysosome-autophagy systems contribute to better proteostasis and longevity, thus inducing the degradation of anti-cancer factors and worsening of cancer.

## Roles of microbiota disorders in aging and cancers

The intestinal microbiota plays an indispensable role in the degradation of carbohydrates, synthesis of short-chain fatty acids, amino acids, and vitamins [37]. Maintaining a normal composition and proportion of intestinal microbiota is the foundation of the integrity of the epithelial barrier and immune balance. In the process of human aging, the composition of the gut microbiota changes constantly and manifests as a decline in microbial diversity, with a decrease in *Bifidobacterium* and increase in *Clostridium, Enterobacteriaceae, and Enterococci* [38]. In aged animals, researchers have observed decreased genera, indicating an antiphlogistic and butyrate-producing action, as well as increased genera, resulting in the degradation of mucin [39]. These changes can be used to explain aggravated gut inflammation and impaired integrity of the gut epithelial barrier in older individuals.

Recently, researchers have revealed the role of intestinal flora in aging. For instance, in a previous study among centenarians and supercentenarians, there was an abundance of *Christensenella, Akkermansia, and Bifidobacterium* [40]. In addition, researchers have observed an increase in the expression of genes regulating xenobiotic biodegradation and metabolism such as toluene, chlorocyclohexane, and caprolactam, and a reduction in the expression of genes manipulating the carbohydrate metabolism in centenarians and supercentenarians [41]. Therefore, it is evident that certain flora may prolong one’s lifespan. A recent study has also proved this hypothesis, showing that administration of *Akkermansia muciniphila* by oral gavage prolonged the lifespan of progeroid mice [42]. Furthermore, *A. muciniphila* supplementation successfully delayed aging-mediated reduction of colonic mucus thickness [43].

In addition, the intestinal flora possibly intervenes in tumorigenesis and disease progression. Previous studies have indicated differences in the intestinal microbiota between healthy people and patients with cancer, which present as a reduction in the number of beneficial bacteria and an increase in the number of opportunistic pathogens in the gut of patients with cancer [44]. In subsequent experiments, most bacteria were proven to have a positive or negative correlation with cancer.

Evidence shows that *Fusobacterium* stimulates the growth and metastasis of CRC and breast cancer [45-47]. In 2020 year, our team further discovered that Fusobacterium motivated the migration of CRC cells and lung metastasis by upregulating the levels of the long non-coding RNA Keratin7-antisense and Keratin7 [48]. In addition, the main mechanisms that have been explored are also involved in virulence factors, immunoregulation, microRNAs, and bacterial metabolism [49]. In addition, colibactin-producing *Escherichia coli* has been shown to be carcinogenic by suppressing effective T-cell response [50]. Moreover, enterotoxic *Bacteroides fragilis* can accelerate tumor growth by enhancing Treg activity or mediating the long non-coding RNA BFAL1, which depends on the RHEB/mTOR pathway [51]. A recent study has also proposed that seropositivity to seven common *E. coli* and two common enterotoxic *B. fragilis* was closely related to CRC progression, especially that of proximal colon cancers [52]. In addition to an increase in the carcinogenic flora, a decrease in probiotics has also been observed in patients with cancer. Therefore, it is evident that there is a close relationship between chronic colitis and CRC. This year, our team revealed that *B. adolescentis* can suppress DSS-induced chronic colitis by enhancing the protective Treg/Th2 response and promoting intestinal flora remodeling [53]. Moreover, based on test data from some references, *Bifidobacteria* can reduce the occurrence and progression of CRC by downregulating EGFR, HER-2, and COX-2 [54], manipulating microRNAs [55,56], regulating immune response and so on [57]. *Faecalibacterium prausnitzii* can also inhibit the development of breast cancer by downregulating the interleukin (IL)-6/STAT3 pathway [58].

In conclusion, it is evident that aging is always accompanied by a decline in probiotics and an increase in harmful bacteria, which is the driving force behind tumorigenesis and cancer progression.

## Traditional mechanisms: changes in the immune microenvironment during aging and in tumors

During the process of aging, chronic inflammation, attributed to an imbalance in inflammatory response, is the most distinct variation. Recent studies have revealed that aging-related chronic inflammation can also affect the progression of aging. In Drosophila, lower immune deficiency (IMD)/nuclear factor-κB (NF-κB) level can mobilize nutrients and prolong lifespan by inhibiting the immune-endocrine axis, whereas higher IMD/NF-κB levels can result in extensive neurodegeneration and early death by increasing antimicrobial peptides [59]. In humans, C-reactive protein, IL-6, and IL-10 are associated with aging-induced osteoarthritis and cognitive decline in older individuals, presenting as impaired executive function and processing speed [60,61].

Inflammation not only accelerates the process of aging but also supports cancer cells. In recent decades, researchers have shown the role of inflammation in tumorigenesis and cancer progression. Furthermore, NF-κB and IL-6 could support the stemness of some cancer cells or induce chemotherapy resistance [62,63]. NF-κB-activated cyclooxygenase-2 has been shown to regulate the magnitude and selectivity of inflammation induced by Bacillus Calmette-Guérin in bladder cancer [64]. In addition, tumor necrosis factor-α mediates the interaction between gastric cancer cells and activated fibroblasts, possibly contributing to tumor metastasis [65].

In older individuals, the existence of chronic inflammation also alerts the immune system to immediate danger. During the aging process, every part of the immune system degenerates, which is called immunosenescence. From the perspective of innate immune cells, maturation of natural killer (NK) cells and accurate anti-inflammatory effects of macrophages and neutrophils are apparently impaired in older individuals [66-68]. Considering adaptive immunity, bone marrow and thymic involution lead to reduction in the generation of naïve T/B cells and accumulation of effector memory T/B cells. Moreover, the normal capability of accumulated memory B cells is also damaged [69,70]. Previous studies have also revealed that age-related chronic persistent inflammation can transform protective immune cells into various immunosuppressive cells, such as regulatory phenotypes of B cells (Bregs), T cells (Tregs), macrophages, and dendritic cells [71].

Many researchers believe that immunosenescence may also be responsible for the genesis and development of tumors. The escape of NK cells in older individuals caused by impaired maturation accelerates the progression of acute myeloid leukemia and decreases the rate of survival [72,73]. In a separate study on aged mice, subtype remodeling of macrophages, which presented as expanded M2 macrophages in the bone marrow and spleens, was probably carcinogenic [74]. In addition, decreased naïve T cells and increased effector memory T cells would lead to incompetent T cell responses to novel tumor-associated antigens. A previous study on non-small cell lung cancer (NSCLC) showed that CD4+ Tregs were responsible for accelerated tumor growth [75]. Núñez et al. pointed out that cancer invasion in draining lymph nodes was also caused by Treg accumulation [76]. Previous studies have also concluded that tumor immune escape is the consequence of cross-regulation between Bregs and cancer cells [77].

Therefore, the aged systemic microenvironment, especially the accumulation of inflammatory factors and immunosuppressive cells, can accelerate aging anteriorly. This vicious cycle aggravates the imbalance of the inflammatory response and triggers immune escape, which may trigger tumorigenesis.

## Energy metabolism and substance metabolism disorders influence both aging and cancer

Metabolism is one of the most basic characteristics of life, and aging is characterized by receding bioenergetics, which are disorders such as those of lipid synthesis and decomposition and glucose utilization [78]. In the present study, we have summarized the relevance of aging and cancer from the perspectives of systemic energy metabolism and local substance metabolism.

Nicotinamide adenine dinucleotide (NAD+) is a key coenzyme that mediates multiple redox reactions and plays an indispensable and irreplaceable role in energy metabolism [79]. Starting from post-puberty, NAD+ in both sexes is negatively correlated with age because of increased degradation and decreased generation [80,81]. In addition to humans, mice and C. elegans also show an age-dependent decrease in NAD+ [82]. NAD+ levels play a crucial role in mitochondrial homeostasis in various species, and aging-mediated absence of NAD+ impairs mitochondrial function [83]. Several aging models have shown mitochondrial fission and fusion which can regulate metabolic efficiency to adapt to rapid changes in nutrient availability were impaired in older individuals. Furthermore, decreased mitochondrial trafficking has also been observed in the process of aging [84].

Subsequently, decreased levels of NAD+ and mitochondrial dysfunction expedite the aging process. Werner syndrome is a premature aging disease characterized by damaged mitochondria and NAD+ deficiency. NAD+ repletion can neutralize premature aging symptoms by restoring mitochondrial function and mitophagy [85]. Moreover, failure in NAD+ signaling has been proven to have a causal relationship with the vascular [86], immune system [87], and nervous system [88], retina [89], cardiac and skeletal muscles during aging [90]. NAD+ can regulate circadian reprogramming to delay aging by suppressing the clock repressor PER2 [91]. However, although NAD+ repletion seemed to counteract the aging process, this method could only improve healthspan in normal individuals. Some studies in mice revealed that their lifespan could not be extended or could only be slightly extended (approximately 40 days) by replenishing NAD+ levels [92,93].

In addition, numerous studies have suggested that NAD+ and mitochondrial dysfunction are closely related to cancer. The key enzymes of NAD+, such as de novo synthesis-nicotinate phosphoribosyltransferase and salvage synthesis-nicotinamide phosphoribosyl-transferase, are overexpressed in various cancers and contribute to higher glycolytic activity, cancer progression, chemoresistance, and poor prognosis [94-97]. According to Chowdhry et al., the survival of tumors relies on two key enzymes [98]. Moreover, a review summarized the role of NAD+ in cancers and concluded that NAD+ was the initiator of the Warburg effect in cancers [99]. Therefore, NAD+ mainly acts as a carcinogenic factor. Although NAD+ is upregulated in cancers, mitochondrial damage and dysfunction such as increased mitochondrial fission, reduced mitochondrial fusion [100,101], mutation and depletion of mitochondrial DNA [102-104], disruption of mitochondrial protein [105,106] and other processess were also universally observed in various cancers which causing cancer growth, metastasis, invasion, and therapeutic resistance.

Regarding local substance metabolism, a previous study conducted by Gomes et al. revealed that the levels of three metabolites—phosphoenolpyruvate (PEP), quinolinate, and methylmalonic acid (MMA) were significantly increased in older individuals compared to those in younger individuals [107]. MMA is a by-product of propionate metabolism that promotes a pro-aggressive epithelial-to-mesenchymal transition-like phenotype by upregulating SOX4 and triggering cancer progression [108]. This may be the most recent cogent evidence to prove the relationship between aging and cancer in terms of substance metabolism. Quinolinate, a metabolite of the kynurenine pathway, participates in NAD+ de novo synthesis. Quinolinate phosphoribosyltransferase is the rate-limiting enzyme in the kynurenine pathway, which is overexpressed in breast cancer and promotes cell migration and invasion [109,110]. In contrast, PEP seems to have anticancer roles. In melanoma, increased PEP bolstered effector functions of tumor-specific T cells, leading to inhibited tumor growth and prolonged survival in rats [111]. This demonstrates that accumulated metabolites during aging may have positive or negative impacts on cancer cells and that the final effect is differ based on tumor types.

In addition, α-ketoglutarate (α-KG), a tricarboxylic acid cycle intermediate, also changes on aging, thus intervening in the processes of both aging and cancer. During the process of aging, Chin et al. reported that α-KG delayed aging and prolonged longevity by approximately 50% by suppressing ATP synthase and decreasing oxygen consumption [112]. The function of α-KG in extending the lifespan has also been observed in Drosophila [113]. Last year, Shahmirzadi further proved that replenishing α-KG not only reduced frailty but also prolonged the lifespan of both female and male mice (especially female mice) by inhibiting aging-related chronic inflammation [114]. α-KG can also delay aging-related osteoporosis and fecundity reduction [115,116].

The effects of α-KG on cancers have also been reported in recent studies. For instance, α-KG supplementation or accumulation can switch glucose metabolism to oxidative phosphorylation from glycolysis, resulting in the inhibition of the Warburg effect, improved tumor suppressors, decreased HIF-1α levels, or downregulated matrix metalloproteinase 3 to suppress breast cancer oncogenesis, progression, and metastasis [117,118]. In CRC, dietary α-KG can regulate the immune system or intestinal flora to inhibit inflammation-related CRC [119]. In contrast, α-KG enhances glucose uptake, sustains cancer cell survival, and accelerates gliomagenesis by activating the IKKβ and NF-κB signaling pathways [120].

## Endocrine hormones may delay aging but promote cancer

Aging is accompanied by a recession of the endocrine axis, which contributes to neuroendocrine disequilibrium, manifesting as hormonal absence or excess. Previous studies have reported a reduction in the levels of thyroid stimulating hormone (TSH), growth hormone (GH), luteinizing hormone (LH), and follicle-stimulating hormone (FSH) in older individuals compared to those in younger individuals with the same health status [121-123].

Recently, hormones (TSH, GH, LH, and FSH) have been found to intervene in the lifespan of an individual. Researchers have revealed that the macrobian population is characterized by high TSH generation and TSH insensitivity without altered energy metabolism [124,125]. Moreover, TSH can reverse aging mediated by BRAFV600E mutations in mice, both in vitro and in vivo [126]. Similar to GH, insulin/insulin-like growth factor I signaling suppression delayed stress granule protein accumulation and subsequently prolonged longevity in C. elegans [127,128]. Furthermore, GH treatment reduced longevity in aged mice (common mice or macrobian mice-Ames dwarfs) by intervening with the genotype, thus confirming the influence of hormones on lifespan [129,130]. In humans, GH receptor exon 3 deletion homozygotes can prolong the lifespan of males by approximately 10 years [131]. In addition, gonadotropin-releasing hormone, which regulates the FSH/LH ratio, has also been proven to restore impaired neurogenesis and delay aging in mice [132].

The relationship between hormones (TSH, GH, LH, and FSH) and cancer has also been previously reported. Abnormally activated TSH receptors can result in accelerated angiogenesis and deterioration of thyroid cancer [133]. Furthermore, high levels of TSH are associated with an increased risk of thyroid cancer [134]. In addition, TSH can promote the growth of ovarian and liver cancers, resulting in poor survival rates and resistance to chemotherapy [135]. Moreover, GH treatment or receptor stimulation increases cancer incidence and the risk of cancer mortality, induces chemoresistance and metastasis in some specific populations and tumors [136-143]. Notably, people or rodents with developmental GH/IGF-1 show rare-to-no cancer occurrence due to the overexpression of DNA repair genes and perpetual strengthening of DNA repair capacity [144]. This phenomenon further confirms the powerful effects of GH/IGF-1 on the promotion of cancers. Regarding gonadotropic hormones, FSH upregulates ACTL6A, a novel oncogene that supports glucose metabolism and, thus, increases the invasiveness of ovarian cancer cells [145].

Therefore, aging is always related to endocrine disorders, which can result in an array of symptoms and diseases; hormone replacement therapy occasionally delays aging. However, direct replenishment of hormones is not appropriate because the method appears to be carcinogenic. In conclusion, the present study clearly indicates that hormones are a link between aging and cancer.

## Similarity in epigenetic changes between aging and cancers

Epigenetics is a branch of genetics that deals with heritable changes in gene expression without altering the nucleotide sequence. Epigenetic phenomena include DNA methylation, genomic imprinting, maternal effects, gene silencing, and RNA editing. Herein, we have discussed the epigenetic phenomena involved in aging and cancer.

Aging is accompanied by constantly decreasing rates of methylation on a genomic level, which incurs chromosomal instability. Moreover, due to this ascending rate of methylation in CpG island, which results from hypermethylation around the promoter of vital genes, these vital genes will gradually be silenced [146-148]. Recent studies have revealed a close association between DNA methylation age and chronological age. For instance, in embryonic stem cells, the age of DNA methylation was approximately zero, and in centenarians, it was consistent with the venerable age (r = 0.89) [149,150]. Acceleration of the DNA methylation age, in other words, DNA methylation age is older than the chronological age, can improve mortality without being influenced by other risk factors [151]. Furthermore, several studies have revealed that individuals live longer, and their offspring exhibit a deceleration in DNA methylation age [152,153].

Similarly, acceleration of the DNA methylation age also exists in cancer. Previous studies have shown that epigenetic age in cancer tissues increases by 40% in the same person compared that in normal tissues and that the acceleration was significantly conspicuous by nearly 36.2 years on average [154,155]. Several studies have proposed that the acceleration of DNA methylation age increases the risk of malignant tumor [156-159]. Furthermore, recent studies have proposed that the level of DNA methylation promoter in body fluids can serve as an early monitoring index in bladder cancer [160], and lung cancer [161] and some other cancers [162,163].

Genomic imprinting is characterized by monoallelic expression. A previous study reported that the area of methylation on IGF2 promoters expanded during the aging process and that the initially unmethylated allele was methylated. This study also pointed that similar expansive methylation of IGF2 promoters was also revealed in some cancers, such as colon and lung cancers [164]. In a related study by Fu et al., IGF2 exhibited a tissue-specific loss of imprinting with aging in the prostate lobes of humans and rats, which may also exist in prostate cancer [165]. RasGrf1 is a paternal imprinted gene, and erasure of its imprinting leads to the downregulation of RasGrf1 and prolonged lifespan in mice [166]. Alterations in imprinting of the RasGrf1 gene is also observed in cancer cells. Furthermore, increased methylation of RasGrf1 in the gastric mucosa of patients with gastric cancer has been observed as compared with healthy individuals, which results in a low expression of RasGrf1 as well as tumor invasion [167].

Heterochromatinization is a method of transcriptional gene silencing. In yeast and Drosophila melanogaster, loss of gene silencing occurs with age, and this phenomenon results from the loss of repressive heterochromatin during aging [168]. Instability or defects within heterochromatin are widespread alterations in cancer, which also take responsibility for the imbalanced epigenome of tumors [169].

## The deepest connection: genetics of aging and cancers

*Klotho*, a human gene located on chromosome 13, has been dubbed the “master of mortal longevity.” *Klotho* produces two proteins by selective splicing: a membrane protein and a circulating protein. Numerous investigations have established that *Klotho* is closely related to aging. The secretion of *Klotho* has been shown to decrease considerably in the kidneys and serum of aged mice [170]. Additionally, a previous study discovered that between the ages of 21 and 39 years, *Klotho* expression decreases at a rate of 0.082 K/S per year in granulosa cells and 31.95 pg/mL per year in the serum [171].

Recent research has demonstrated that aging-related *Klotho* depletion accelerates aging. Chen discovered that mutations in the *Klotho* gene resulted in the suppression of the transcription factor Nrf2 and inactivation of glutathione reductase, resulting in cardiac aging [170]. Xiaofei Xu et al. demonstrated an intimate relationship between low *Klotho* expression and impaired ovarian reserve [171]. Ullah et al. proposed that *Klotho* deficiency impaired telomerase activity, resulting in stem cell aging and apoptosis [172]. Additionally, it has been demonstrated that the *α-klotho* protein fragment successfully improves cognitive competence and neural resilience in aged mice [173].

Additionally, *Klotho* acts as a tumor suppressor, disrupting neoplastic glycolysis and mitochondrial function while sparing normal cells [174]. Rubinstein et al. pointed out that treatment with *klotho* or its active region KL1 could inhibit CRC growth in vivo or in vitro by improving the unfolded protein response [175]. *Klotho* also inhibits the NF-κB pathway and suppresses CCL2 transcription, thereby decreasing CRC progression [176]. *Klotho* functions as an IGF-1R inhibitor in diffuse large B-cell lymphoma, inhibiting cell apoptosis and proliferation [177].

Additionally, there are numerous other common genetic factors associated with aging and cancer. However, it is important to note that some antiaging genes are bidirectional.

*AUF1* encodes RNA-binding proteins. Pont et al. postulated that *AUF1* activates the telomerase promoter and assumes responsibility for the maintenance of telomere length. *AUF1*-deficient mice would present with premature aging [178]. Additionally, He et al. confirmed that *AUF1* can prevent aging in vascular endothelial cells [179].

*AUF1* has also been shown to affect cancer cells; this regulatory effect is bidirectional. *AUF1* induces apoptosis and suppresses cell proliferation by inhibiting the proto-oncogene bcl-2 and cyclin D1. Its presence plays a critical role in the antitumor activity of certain tumor suppressors, such as p63 [180]. In contrast, the overexpression of *AUF1* has been shown to promote malignant transformation and cancer progression [181]. For example, *AUF1* accelerates the development of CRC by activating the ERK1/2 and AKT pathways and is associated with a poor prognosis [182]. Additionally, it promotes the proliferation and invasion of breast cancer and thyroid cancers and results in poor outcomes in patients with hepatocellular carcinoma [183,184].

The turning point of this bidirectional effect may be the level of expression. In other words, a normal expression of antiaging genes ensures that anticancer pathways remain functional. However, aberrant or obligatory overexpression results in retroaction by excessive activation of tumor promoters.

*SIRT1* is downregulated in aging individuals and plays a role in delaying the onset of organic aging [185,186]. Simultaneously, *SIRT1* acts as a protective gene in certain types of cancers, including gastric and breast cancer, and its deficiency accelerates cancer progression and chemoresistance. Therefore, *SIRT1* overexpression may inhibit the proliferation of cancer cells [187,188]. However, this protective effect is not universal. In certain cancers, such as CRC and cervical cancer, artificial overexpression significantly enhances proliferation, invasion, and stemness, whereas spontaneous knock-out has an inhibitory effect [189-191].

Therefore, we may also deduce that the effect of particular antiaging genes on various cancers is highly variable, regardless of whether they are expressed passively or silently, and may differ according to tissue specificity.

*p16* is a biochemical indicator of biological senescence that increases with age [192]. Previous studies have established that the downregulation of *p16* expression improves the regenerative capacity of pancreatic β-cells [193] and rescues the degradation of the thymus [194], adipose tissue, skeletal muscle, and eyes [195,196]. In general, *p16* is a pro-aging element.

In contrast to what we have previously stated, this pro-aging factor generally functions as a tumor suppressor gene. *p16*, a cyclin-dependent kinase inhibitor, arrests cells in the G1 phase. Numerous carcinogens, including miR-30 and constitutive photomorphogenesis 9 signalosome 6, cause cancer by suppressing *p16* expression, enhancing cell division, and interfering with efficient cellular senescence [197,198]. Liu et al. also demonstrated that ablation of *p16* in B cells could reverse aging while significantly increasing the incidence of B-cell neoplasms [199]. These findings suggest that cellular senescence may have evolved as a strategy to protect against cancer.

In conclusion, from a genetic standpoint, delaying aging does not always suppress cancer.

## Aging and cancer have common means of interventions

**Figure 3.**
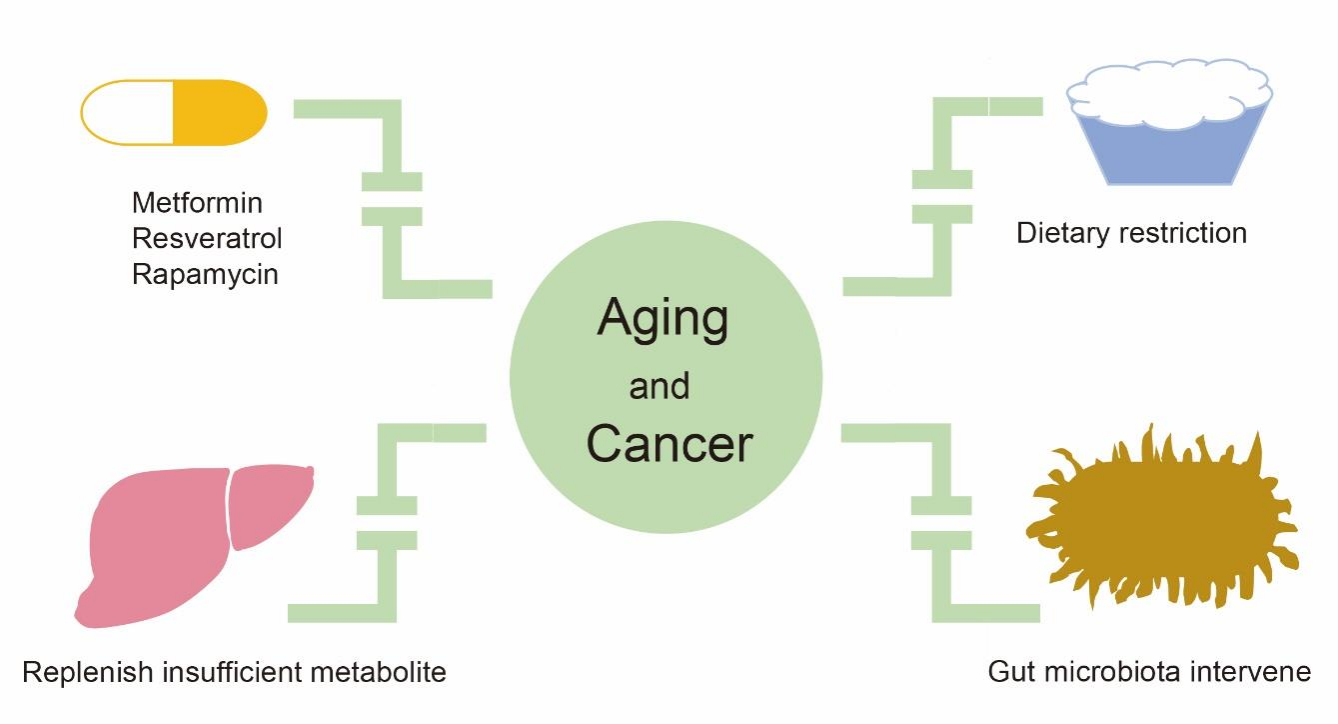
Common means of interventions between aging and cancer.

Antiaging and anticancer agents are two important subjects that compel us to pursue them indefinitely. Here, we summarize the approaches that can delay aging, or even prolong lifespan, and suppress cancer synergistically (Fig. 3,Table 3, and Table 4).

## Repurposing common medicines to treat aging and cancer

While researching antiaging and anticancer treatments, researchers surprisingly discovered that some medications possessed dual effects.

### 1. Metformin.

In mice, metformin delayed ovarian and gut aging, attenuated hearing loss and age-related neuro-degeneration, and improved skin healing abilities in aged rats [200-204]. In inbred 129/Sv mice, Anisimov et al. discovered that treatment with metformin reduced the mean lifespan by 13.4% in males, but increased it by 4.4% in females [205]. However, another study found that the opposite phenomenon of metformin supplementation in the neonatal period extended the mean lifespan (+20%) and maximum life span (+3.5%) in male mice rather than in female mice [206]. Zhu et al. further showed that metformin impaired cardiac homeostasis and longevity in female mice [207]. Therefore, the sex-related differences in the antiaging effects of metformin are significant and warrant further investigation. In humans, metformin delayed the aging of mesenchymal stem cells and reversed mitochondrial function in fibroblasts derived from patients with parkinsonism [208,209]. Additionally, metformin improves muscle strength and alleviates frailty syndrome in the elderly [210]. In terms of cognitive competence, metformin has been shown to delay cognitive deterioration in diabetic older adults [211]. An ongoing study sponsored by the Albert Einstein College of Medicine is delving deeper into the antiaging properties of this incredible drug. They compared the number of upregulated genes in the muscle and adipose tissue of young healthy subjects categorized into two groups (metformin treated and placebo treated) after the intervention, which lasted 6 weeks, and discovered that the metformin group had significantly fewer genes than the control group, regardless of muscle or adipose tissue. This finding suggests that metformin may alter biology at the genetic level. By examining the disparate genetic functions, researchers will have a better understanding of the antiaging mechanisms of metformin (https://clinicaltrials.gov/ct2/show/NCT02432287).

To date, numerous studies have been conducted on the anticancer properties of metformin. Metformin has been shown to suppress tumorigenesis, growth, metastasis, and improve chemosensitivity in various cancers in both in vitro and in vivo animal experiments [212-216]. In humans, a recent trial demonstrated that treatment with metformin could improve disease-free survival (not with metformin: HR = 1.40, p = 0.043), distant disease-free survival (not with metformin: HR = 1.56, P = 0.013), and overall survival (not with metformin: HR = 1.87, p = 0.004) in patients with diabetes and HER2-positive breast cancer [217]. Additionally, another phase 2 trial indicated that metformin supplementation increased cisplatin sensitivity and improved overall survival compared to the expected survival in nondiabetic patients with ovarian cancer [218]. Metformin possesses anticancer properties in NSCLC [219]. However, a trial of patients with advanced pancreatic cancer discovered that the administration of metformin did not improve the prognosis of patients treated with chemotherapy [220]. Therefore, we can deduce that the anti-cancer effects of metformin are unaffected by diabetes mellitus but vary according to tumor type.

Most importantly, Anisimov et al. observed that treatment with metformin initiated at a young and middle-age prolonged longevity (young: +14.1%; middle: +6.1%, p > 0.05) and delayed tumorigenesis (young: delay by 22%; middle: delay by 25%) in female SHR mice. Correspondingly, commencing metformin treatment at an advanced age did not increase life expectancy and lost its anticancer effects [221]. Moreover, in 129/Sv mice, metformin extended the mean lifespan of healthy males (+20%, p< 0.05) and tumor-bearing males (+4%, although p = 0.177). In contrast, it was unable to slow aging in healthy females, while simultaneously improving survival in tumor-bearing females [206]. These phenomena could be explained in part by the putative associations between anticancer effects and the antiaging properties of metformin.

**Table 3:** The effect of different therapies in rats.

Intervention	Prolong lifespan	Inhibit cancer	Both in a model
**Medicine**			
Metformin Resveratrol Rapamycin	Yes (sex specificity) [205-207]	Yes [212,215,216]	Yes [205,206,221]
	Yes [226-228]	Yes [236-239]	No evidence
	Yes (sex specificity) [244-246,351]	Yes [248,250]	Yes [255,256,351-354]
**DR**			
**CR**	Yes [264,265]	Yes [268-270]	Yes [272,273]
**KD**	Yes [277]	Yes [279-282]	Yes [277]
**Microbiota**			
**Symbiotic**	Only delay aging[286-289]	Yes [295-298]	No evidence
**FMT**	Yes [302]	Yes [304-307]	No evidence
**Metabolites**			
**NMN**	Only delay aging[313-319]	No evidence	No evidence
**Spermidine**	Yes [327,330,331]	Yes [341,342]	Yes [331]

DR, dietary restriction; CR, caloric restriction; KD, ketogenic diet; FMT, fecal microbiota transplantation; NMN, nicotinamide mononucleotide.

### 2. Resveratrol

In mice, resveratrol therapy delays aging of the cardiovascular system, improves cognitive performance, and reduces aging-mediated renal impairment [222-225]. Additionally, it increased the life expectancy of HtrA2 knockout mice (mitochondrial dysfunction), SOD1G93A transgenic mice (amyotrophic lateral sclerosis), and SAMP8/1 mice (Alzheimer’s disease) [226-228]. In humans, resveratrol treatment has been shown to slow the process of skin aging [229], improve cerebrovascular function, delay cognitive decline [230,231], and improve mobility-related physical function [232].

The anticancer properties of resveratrol have also been investigated. In an in vitro experiment, resveratrol successfully inhibited the migration, invasion, and viability of cancer cells and induced cell death [233-235]. In vivo experiments showed that resveratrol suppressed tumorigenesis, decreased micro-vessel formation of tumors, alleviated tumor burden, and improved chemotherapeutic response in mice [236-239]. Unfortunately, clear and direct evidence regarding the effects of clinical treatment continues to be insufficient.

### 3. Rapamycin

Short-term rapamycin treatment inhibited premature ovarian failure, rejuvenated oral health, and slowed the epigenetic age of the liver in aging mouse models [240-242]. In terms of lifespan, Bitto et al. observed that 3 months of rapamycin supplementation increased the life expectancy of middle-aged mice by 60% [243]. Strong et al. further pointed out the sex-specific effects of rapamycin, stating that a 3-month course of rapamycin improved longevity in male mice, whereas female mice required continuous exposure [244]. However, it has been demonstrated that rapamycin does not extend longevity in progeroid models with DNA repair deficiency or telomerase deletion, regardless of dosage, gender, or timing [245,246]. This indicates that the antiaging effects of rapamycin are sex-specific and that its mechanism of action may involve enhancing DNA repair capacity and preventing DNA damage. Topical rapamycin treatment delays skin aging and improves skin appearance in humans [247].

In terms of anticancer activity, rapamycin alone or in combination with other agents has been shown to decrease the progression of various tumors or to enhance the chemotherapeutic response in vitro or in vivo [248-250]. Clinically, as an adjuvant therapy, rapamycin successfully developed a pathological response and inhibited metabolic activity in rectal cancer [251], suppressed head and neck tumor growth [252], enhanced the curative effect of intravesical BCG therapy [253], decreased the incidence of secondary skin cancers, and improved squamous cell cancer-free survival at 5 years (p = 0.007) in kidney transplant recipients [254]. Of course, these are phase I and II trials, and additional clinical trials are needed to investigate the direct therapeutic effects of rapamycin in various types of cancers.

Several studies on rats have demonstrated a more direct connection between aging and cancer. For example, Anisimov et al. discovered that rapamycin treatment increased the mean (+4.1%) and maximal lifespans (+12.4%), as well as delayed the incidence of breast cancer (p < 0.001) and decreased tumor burden (p < 0.01) [255]. Wilkinson et al. reported that rapamycin delayed the aging of multiple systems while decreasing the incidence rates for adrenal tumors (p = 0.04) and lung tumors (no statistical significance) [256]. Additionally, researchers discovered that rapamycin, despite its ability to prevent cancer growth, failed to prolong survival in rats already suffering from tumors. Therefore, we conclude that rapamycin suppresses tumorigenesis by delaying aging rather than delaying death by inhibiting cancer. Hence, there are several targets controlled by rapamycin in cancer and aging.

**Table 4:** The effect of different therapies in humans.

Intervention	Delay aging	Prolong lifespan^1^	Inhibit cancer	Improve prognosis
**Medicine**				
Metformin Resveratrol Rapamycin	Yes [208-211]	No evidence	Yes (vitro and vivo) [212-214,216]	Yes [217-219]
	Yes [229-232]	No evidence	Yes (vitro) [233-235]	No evidence
	Yes [247]	No evidence	Yes (vitro and vivo) [248,249,251-254]	No evidence
**DR**				
**CR**	Yes [266]	Yes^2^[267]	Yes (vivo)^2^[271]	No evidence
**KD**	Yes [278]	No evidence	Yes (vivo) [284]	Yes [284,285,355]
**Microbiota**				
**Symbiotic**	Yes [290-294]	No evidence	Yes (vivo)^2^[299,301]	Yes^3^[300]
**FMT**	No evidence	No evidence	Yes (vivo) [308]	Yes [308]
**Metabolites**				
**NMN**	No evidence	No evidence	Unclear [320,321]	No evidence
**Spermidine**	Yes [332-334]	Yes^2^[335]	Yes (vitro and vivo^2^) [336-340]	No evidence

DR, dietary restriction; CR, caloric restriction; KD, ketogenic diet; FMT, fecal microbiota transplantation; NMN, nicotinamide mononucleotide.

1 Prolong lifespan means that this method can extend longevity of whole populations rather than patients with specific diseases.

2 Only proved in retrospective study.

3 0.05 < P-value < 0.1

## Conserved role of dietary restriction in aging and cancers

In recent years, we have begun to pay more attention to dietary restriction which has been shown to extend the healthy lifespan of several species without causing malnutrition. Caloric restriction (CR), restriction of specific dietary ingredients, ketogenic diet (KD), fasting, and so on are all examples of dietary restriction [257]. In this study, we were mostly concerned about the role of CR and KD in aging and cancer.

### 1. CR

In rats, CR restored redox homeostasis in aged hearts [258], delayed skeletal muscle degeneration [259], postponed aging-correlative recession in locomotor activity [260], alleviated aging-related metabolic disorders [261], remodeled age-related methylation pattern[262], and even caused epigenetic age deceleration [263]. In terms of longevity, in the last century, people have observed that 40% of long-term CR maintains cellular replication capacity and increases both average lifespan and maximum lifespan by 36% and 20%, respectively [264]. Yan et al. demonstrated that lifelong CR increased the survival of rats by 30% when combined with GH/IGF-1 suppression, while GH/IGF-1 suppression alone only improved survival by 8% [265]. In humans, CR prolonged the replicative lifespan of adipose-derived stromal/progenitor cells and delayed white adipose tissue recession [266]. A retrospective study revealed that Okinawans who adhered to a CR diet extended both average and maximum longevity compared to Japanese and Americans [267].

In terms of anticancer effects, CR has been shown to decrease the progression of numerous cancers and improve the therapeutic response in mice [268-270]. Additionally, clinical studies have shown that CR successfully reduces the incidence of breast cancer (all women: OR = 0.52, p = 0.001; premenopausal women: OR = 0.36, p < 0.001; postmenopausal women: OR = 0.77, p = 0.001) [271].

The antiaging and anticancer effects of CR were observed using the same models. Weindruch et al. discovered that CR not only extended the lifespan of mice but also suppressed the occurrence of spontaneous lymphoma [272]. Lee et al. found that CR prolonged the maximum lifespan of mice by 13% (p < 0.0001) and appeared to decrease the incidence of hepatocellular carcinoma and lymphoma in mice (both p ≤ 0.001) [273].

### 2. KD

KD is a low-carbohydrate diet with a high fat content (5-10% of the total daily consumed kcal). Recently, it was discovered that non-obesogenic KD improved memory [274], inhibited myocardial remodeling and damage [275], and preserved skeletal muscle mass and function in mice during the aging process [276]. Additionally, Megan et al. demonstrated that KD preserved physiological function while increasing median longevity by 13.6% along with survival in mice [277]. Numerous investigations conducted in humans have established that KD improves cognitive function and memory in patients with Alzheimer’s disease, particularly those with the APOE4^-^ genotype, even in short-term memory [278].

The anticancer effects of KD were also investigated. For example, in a mouse model, KD alone has been shown to inhibit the growth of a variety of tumors [279,280]. In mice with pancreatic cancer, combining radiotherapy with KD may improve radiation sensitivity and extend survival time compared to radiotherapy alone [281]. Additionally, it may enhance the anticancer properties of rapamycin and other chemotherapeutic agents [279,282]. In humans, KD has been shown to enhance the effects of chemotherapy, promote the incidence of complete or partial responses, and increase patient survival rate without inducing side effects [283-285]. In summary, KD serves as an adjuvant therapy to enhance the effectiveness of conventional therapy.

It is worth mentioning that KD not only increased median lifespan by 13.6% in a mouse model, but also decreased the incidence of malignancies, particularly histiocytic sarcoma (P < 0.1), at the time of death. This effect was dose-dependent [277]. This result further established that energy and substance metabolism connect aging with cancer, which is likely to be mediated by mitochondrial function and oxidative stress.

## Emerging approaches: interventions in the gut microbiota in aging and cancer

Previous research has established that disturbances in the intestinal microbiota occur inexorably with aging and can result in various age-related diseases, including cancer. Thus, microbiota intervention strategies such as probiotics, prebiotics, symbiotics, and fecal microbiota transplantation (FMT) are emerging to rectify flora disturbances.

### 1. Symbiotic

Symbiotic bacteria have been shown to increase cognitive function, minimize memory deterioration, delay behavioral degeneration (such as muscular vigor and exploratory activity), and protect against primary and secondary osteoporosis in aging mice [286-289]. In humans, earlier research concluded that symbiotic bacteria restore the skin barrier; protect against photoaging; and improve hair quality [290], cognitive function, and aging-mediated memory impairment during aging [291-293]. Liu et al. also argued that ingesting symbiotics significantly upregulated serum calcium concentrations in older individuals, although its benefits to bone health remained ambiguous [294].

Recently, a plethora of evidence supporting the anticancer effects of symbiotics has emerged. Previous research has shown that symbiotics, such as *Bifidobacterium, Bacteroides fragilis*, and *Akkermansia muciniphila*, can inhibit cancer progression, mainly breast and CRC, and enhance chemotherapy effects in vitro or in mice [295-298]. Clinically, Toi et al. discovered that *Lactobacillus casei strain Shirota* consumption showed a negative correlation with breast cancer incidence (OR = 0.65, p = 0.048) [299]. Takada et al. demonstrated that supplementation with probiotics improved progression-free survival (HR = 1.73, p = 0.0229), disease control (OR = 0.51, p = 0.0004), and overall response (OR = 0.43, p < 0.0001) in NSCLC patients receiving anti-programmed cell death 1 protein (anti-PD-1) monotherapy [300]. Additionally, the use of probiotics decreased the incidence of metachronous gastric cancer after endoscopic resection (HR = 0.29, p = 0.034) [301].

### 2. FMT

It is worth noting that FMT from wild type mice prolongs the life of progeroid mice, and it has been established that *Akkermansia muciniphila* plays a predominant role. Antiaging effects may be dependent on secondary bile acid restoration [302]. Additionally, another study discovered that FMT was capable of successfully regulating Hippo signaling, which is involved in the manipulation of aging processes [303].

An earlier study showed that FMT from normal mice to tumor-bearing mice decreased tumor load and size in the azoxymethane-dextran sodium sulfate (AOM-DSS) model of CRC [304,305]. Gopalakrishnan et al. observed that the gut microbiome of patients responding to anti-PD-1 could improve antitumor immunity in germ-free mice and restore the effects of PD-1 blockade in resistant mice [306,307]. In humans, a recent clinical study discovered that FMT from healthy obese individuals to patients with gastroesophageal cancer improved therapeutic response (p = 0.035), and may also improve overall survival (HR = 0.38, p = 0.057) and progression-free survival (HR = 0.5, p = 0.092) in patients [308].

The use of gut microbiota intervention for anti-aging and anti-cancer strategies is currently limited. Additional evidence is necessary to support this therapy.

## Replenishing insufficient metabolites: is it effective and safe enough?

Previous research has shown that circulatory metabolites, including NAD+, glutathione, spermidine, glutamine, and α-ketoglutarate, decrease significantly in the older individuals [309-312]. Therefore, the role of restoring these depleted metabolites in antiaging processes is of interest. Here, we have focused on nicotinamide mononucleotide (NMN) and spermidine.

### 1. NMN

NMN, as an NAD+ booster, has been shown to delay vascular aging, promote functional vascular rejuvenation, and reverse vascular dysfunction in aged mice [313,314]. It also alleviates memory recession and improves learning and cognitive function in mice [315-317]. Additionally, NMN supplementation has been shown to enhance osteogenesis and improve bone density, locomotor activity, and eye function in aged mice [318,319]. However, the direct antiaging effects of NMN have not been elucidated in humans yet.

With regard to cancer, NAD+ is catalyzed by nicotinamide mononucleotide adenylyl transferases (NMNATs, the rate-limiting enzymes) from NMN. In humans, earlier research found that NMNAT2 is highly expressed in CRC and its increased expression correlates with a deeper invasive depth and an advanced TNM stage (p < 0.05) [320]. Additionally, as previously stated, NAD+ is a known carcinogen in a wide variety of malignancies. NMN supplementation appears to be involved in the progression of cancer. However, Grozio et al. discovered that increased expression of SLC12A8, a particular NMN transporter required for NMN uptake, was associated with a favorable prognosis in patients with pancreatic and breast cancer [321]. Zong et al. discovered that in a mouse model, NMN supplementation could prevent liver fibrosis, which could progress to liver cancer [322].

However, the effects of NMN on cancers are unknown and it is unclear whether replenishing NMN for antiaging purposes is carcinogenic.

### 2. Spermidine

Recent research in aged mice discovered that dietary spermidine can improve structural brain measures [323], improve spatial and temporal memory [324], decrease locomotor activity loss [325], protect cardiac function [326,327], delay skeletal muscle atrophy [328] and reverse arterial aging [329]. In terms of lifetime, it has been observed that dietary polyamines (spermine and spermidine) prolong longevity and improve survival in ICR mice [330]. Eisenberg et al. further reported that late-life spermidine replenishment (which is easier to achieve in humans) successfully improved the median lifespan by 10% [327]. Yue et al. pointed out that lifelong spermidine supplementation can increase the lifespan of mice by 25% [331]. In humans, spermidine has been shown to preserve female fertility [332], restore skin structure and barrier function [333] and improve mnemonic discrimination performance [334]. In humans, spermidine has been shown to preserve female fertility [335].

Similarly, spermidine was found to decrease cervical cancer in vitro by suppressing HeLa cell proliferation and inducing apoptosis intrinsically or via an apoptosis-inducing factor [336-338]. Razvi et al. discovered that acylspermidine derivatives inhibited breast and blood cancer cell proliferation and triggered apoptosis [339]. Additionally, the presence of spermine/spermidine could induce apoptosis in CRC cells mediated by maize polyamine oxidase [340]. In mice, spermidine inhibited the growth of CT26 CRC cells transplanted into immunocompetent individuals [341]. Soda et al. also discovered that spermine and spermidine intake decreased the incidence of colon cancer induced by a chemical carcinogen in BALB/c mice [342]. Vargas et al. discovered that spermine and spermidine could significantly decrease the risk of CRC in women with a body mass index of ≤25 (HR = 0.58, p = 0.012) or in women who consume more fiber than the average (HR = 0.44, p = 0.015) [343].

Most importantly, Yue et al. demonstrated that spermidine not only extended the lifespan but also alleviated liver fibrosis and inhibited hepatocellular carcinoma by improving MAP1S-mediated autophagy in the same model. This phenomenon indicated that spermidine could regulate both aging and cancer synergistically, possibly by manipulating autophagy [331].

## Discussion

Aging is an inevitable process. Economic, technological, and medical advancements have resulted in a decline in birth rates and an increase in the proportion of the older individuals, putting a strain on the health system. The increased incidence of various diseases in aged individuals implies that aging impairs health. As one of the leading causes of death among the elderly, a higher diagnostic rate of malignant tumors in the elderly also suggests a possible link between cancer and aging. For example, in the United States, more than 60% of all lung cancer cases occurred in those aged more than 65 years, whereas less than 2% of lung cancer occurred in people younger than 45 years [344]. Hospitalization costs associated with anticancer treatments have increased significantly in recent years. To extend a healthy lifespan and alleviate the strain on the health care system, it is critical to explore the link between aging and cancer. With the advancement of scientific research, significant changes in the human body during the process of aging have already been identified, including chronic inflammation, immunosuppression, and endocrine disorders. Additionally, numerous pathogenic mechanisms for common cancers have been proposed, and we identified some aging-related alterations associated with tumor occurrence. Worse yet, these age-related alterations might accelerate the rate of aging, creating a vicious circle. Hence, cancer therapy targeting aging is likely to be effective.

During the last century, researchers have already noticed a decreased in the incidence of malignant tumors in patients with Huntington’s disease, which may be attributed to accelerated programmed cell death of precancerous cells [345]. Several years ago, Musicco et al. discovered that older individuals with Alzheimer’s disease had a lower risk of developing malignant tumors [346]. These findings appear to support a negative correlation between cancer, proliferative disease, and aging, which is typically associated with degenerative diseases.

However, this correlation was not statistically significant. Last year, Ording et al. conducted additional research on the correlation between Alzheimer’s disease and cancer. They established that the negative correlation between these two diseases was quite limited (SIR = 0.94, 95% confidence interval 0.92-0.96) and weakened over time [347]. Freedman et al. found no correlation between site-specific cancers and amyotrophic lateral sclerosis [348]. Liu et al. discovered a statistically significant positive correlation between the occurrence of melanoma and Parkinson’s disease [349].

The association between cancer and degenerative diseases does not always exhibit an inverse correlation; it can occasionally exhibit a positive correlation. This suggests that the relationships between cancer and aging, or organic aging, are intricate, and their tissue specificity should be investigated.

In this review, we summarized numerous strategies for delaying aging and restoring aging-related imbalances or disorders. Additionally, these techniques have been shown to have anticancer effects. At the same time, we further summarized the experimental phases of these strategies to estimate their feasibility and reliability.

Metformin is a relatively well-studied medication. Researchers have established that it can slow organic aging and ameliorate the symptoms of senile diseases. It may also act as a regulator of human genes, bringing their expression levels closer to those of young people (https://clinicaltrials.gov/ct2/show/NCT02432287).

Cancer has the potential to suppress tumor progression both in vivo and in vitro, as well as improve patient prognosis. However, its anticancer or antiaging effects may be gender- and tissue-specific, which have not been adequately investigated. Resveratrol and rapamycin have also been shown to delay organic aging and slow tumor progression; however, evidence of improved prognosis in cancer patients is currently lacking. Dietary restriction has already been shown to have anticancer and antiaging properties, which has been partially verified in humans. Additionally, there is a significant problem in that the level (percentage) or pattern (persistent or intermittent) of restriction, the species of restricted energy, and age or gender disparity are ambiguous. The effects of microbiota intervention on aging and cancer have been primarily tested in animals. Owing to concerns regarding the complexity of the methods, the survival rate of xenogenous microflora, and demic tolerance, the application of FMT in the human body is rather uncommon. Thus, its antiaging and anticancer effects must be further validated. Replenishment of scarce metabolites in elderly individuals, such as spermidine, is another attempt at this monumental task that has been demonstrated in animals to a certain extent. However, clinical trials in humans have not yet begun. Additionally, researchers discovered that spermidine, a polyamine, may suppress carcinogenesis but deteriorates established tumors [350]. Its bidirectional characteristics remain obscure. Additionally, all the approaches outlined above lack direct evidence of extending healthy human life and can only be used as adjuvant medications or therapies for cancer treatment.

Hence, one of the most critical objectives of future research will be to optimize interventions for them to be extensively employed in clinical practice. For example, extending the scope of clinical trials based on safety to further evaluate the therapeutic effects and determine the optimal dose. Developing an appropriate dosage or compound that maximizes bioavailability, minimizes side effects, and improves compliance is critical. Additionally, determining the primary and most appropriate target for each individual and developing an individualized treatment plan are necessary before being extensively used in the clinic. Senolysis offers an alternative to the traditional prevention paradigm of “antiaging therapy,” which entails lifestyle or pharmacological interventions to delay the onset of age-induced decrepitude and the occurrence of diseases. The complexity of the human body and the diversity of aging and cancer mechanisms prevent us from conducting extensive and exhaustive research. Thus, additional methods need to be explored. It is notable that blind supplementation with missing substances is not a good idea. In contrast, it may fail to delay aging and even induce tumorigenesis. In the future, we should devote our efforts to precisely identify therapies that would effectively prevent the incidence and progression of cancer by delaying aging.
